# Glasgow Prognostic Score and Gustave Roussy Immune Score in Hodgkin Lymphoma: Survival Associations and Limited Incremental Prognostic Value Beyond the International Prognostic Score

**DOI:** 10.3390/jcm15135159

**Published:** 2026-07-02

**Authors:** Kemal Aygün, Şerife Solmaz, Olgu Aygün, İbrahim Eryılmaz, Tugba Cetintepe, Hatice Demet Kiper Unal, Alev Garip Acar, Eray Arslan

**Affiliations:** 1 Hematology Clinic, İzmir Katip Çelebi University Atatürk Training and Research Hospital, 35170 İzmir, Turkey; 2 Family Medicine, İzmir City Hospital, 35170 İzmir, Turkey; 3 Family Medicine, Amasya Göynücek District State Hospital, 05900 Amasya, Turkey

**Keywords:** Hodgkin lymphoma, Gustave Roussy Immune Score, Glasgow Prognostic Score, International Prognostic Score, progression-free survival, overall survival, systemic inflammation, prognosis

## Abstract

**Background/Objectives:** Although outcomes in Hodgkin lymphoma (HL) have improved substantially, patients with advanced-stage disease, comorbidities, or relapsed/refractory presentations can still fare poorly. Blood-based indices of systemic inflammation and nutrition are derived from routine tests, but their value beyond established prognostic models is uncertain. We examined the association of the baseline Gustave Roussy Immune Score (GRIm) and Glasgow Prognostic Score (GPS) with treatment response, progression-free survival (PFS), and overall survival (OS) in HL, focusing on their performance relative to the seven-factor International Prognostic Score (IPS-7). **Methods:** We retrospectively analysed 110 adults with histologically confirmed HL treated at a tertiary haematology centre between January 2015 and December 2025. GPS, GRIm, and IPS-7 were calculated from data recorded at diagnosis. Treatment response was classified as complete versus non-complete. Outcomes were assessed with Kaplan–Meier analysis, log-rank tests, Cox regression, Harrell’s C-index, and likelihood-ratio testing. **Results:** Most patients had advanced-stage disease (69.1%) and received ABVD-based treatment (94.5%); complete response was achieved in 90 (81.8%). GPS and GRIm were not significantly associated with non-complete response, whereas IPS-7 was. Over a median follow-up of 39.5 months, 28 patients (25.5%) progressed or died and 17 (15.5%) died. In univariable Cox analysis, high GRIm risk (HR = 2.68, 95% CI 1.17–6.14), higher GPS (HR = 2.18 per point, 95% CI 1.23–3.89), and higher IPS-7 (HR = 2.10 per point, 95% CI 1.59–2.77) predicted shorter PFS. For OS, GPS and IPS-7 were significant, whereas GRIm was not. After adjustment for IPS-7, neither GPS nor GRIm remained independently associated with PFS or OS, and adding either score to IPS-7 produced only small, non-significant gains in discrimination. **Conclusions:** Baseline GPS and GRIm were associated with survival on univariable analysis, particularly for PFS, but their incremental value beyond IPS-7 was limited. These scores may help describe baseline inflammatory and nutritional risk and should not be regarded as alternatives to established HL prognostic models. In particular, GPS and GRIm were not significantly associated with treatment response and should be viewed as supportive markers requiring external validation, rather than as tools that can independently guide treatment decisions.

## 1. Introduction

Hodgkin lymphoma (HL) is a B-cell-derived malignancy defined histologically by Reed–Sternberg cells embedded in a reactive inflammatory infiltrate. It represents roughly 10% of all lymphomas and follows a bimodal age distribution, with incidence peaks in young adults and in patients older than 55 years [1]. Modern multi-agent chemotherapy, PET-adapted treatment, and improved supportive care have raised long-term survival above 90% in limited-stage disease [1,2]. Despite this progress, a clinically important minority of patients—those with advanced-stage disease, older age, comorbidities, high tumour burden, or relapsed/refractory disease—still relapse or die, which keeps the search for better baseline risk markers clinically relevant [3,4].

Risk assessment in HL has long relied on clinical and laboratory variables, including Ann Arbor stage, bulky disease, B symptoms, haemoglobin, leucocyte count, and lymphocyte count. These variables underpin the seven-factor International Prognostic Score (IPS-7), which remains the standard reference model in advanced-stage disease [5]. IPS-7 nonetheless leaves substantial outcome heterogeneity unexplained: patients within the same risk category can follow very different clinical courses, so simple markers that capture host inflammatory and nutritional status could, in principle, refine baseline stratification.

Functional imaging with 18F-fluorodeoxyglucose PET/computed tomography is central to staging and response assessment in HL [6], and quantitative measures such as baseline metabolic tumour volume carry independent prognostic weight [7]. These metrics are not yet standardised across centres, however, and are not universally available [8]. Inexpensive indices derived from routine blood tests at diagnosis, therefore, remain attractive, particularly when interpreted alongside—rather than instead of—established prognostic scores.

The Glasgow Prognostic Score (GPS), based on C-reactive protein (CRP) and albumin, summarises systemic inflammation together with nutritional reserve and is prognostic across many solid and haematological cancers [9]. In advanced classical HL, a higher GPS at diagnosis has been reported as an independent predictor of survival [10], and a recent meta-analysis confirmed its prognostic value across haematological malignancies [11].

The Gustave Roussy Immune Score (GRIm) combines the neutrophil-to-lymphocyte ratio (NLR), lactate dehydrogenase (LDH), and albumin. It was originally developed to select patients for early-phase immunotherapy trials [12] and has since shown prognostic value in several solid tumours [13,14,15], but its role in HL is poorly defined. Other routine blood-based markers, including the HALP score and the NLR, have also been linked to outcome in HL [16,17], reinforcing the broader relevance of inflammation- and nutrition-based indices in this disease. Even so, GPS and GRIm have rarely been compared directly within the same HL cohort, and—critically—neither has been formally tested against IPS-7. We therefore evaluated the association of baseline GPS and GRIm with treatment response, PFS, and OS in classical HL, and used IPS-7–adjusted Cox models and model-discrimination analyses to determine whether either score adds prognostic information beyond IPS-7.

## 2. Materials and Methods

### 2.1. Study Design and Population

This retrospective, single-centre study was conducted at the Hematology Clinic of İzmir Katip Çelebi University Atatürk Training and Research Hospital. Adult patients with histologically confirmed HL who received systemic chemotherapy at this institution between 1 January 2015 and 30 December 2025 were reviewed. Clinical, pathological, laboratory, imaging, treatment, response, and follow-up data were obtained from the institutional electronic health record system, including haematology outpatient records, inpatient files, pathology reports, PET/CT reports, chemotherapy protocols, and follow-up notes.

Patients were eligible if they were 18 years or older at diagnosis, had histopathologically confirmed HL, received at least one cycle of systemic chemotherapy during the study period, and had sufficient baseline clinical and laboratory data to calculate the GPS, GRIm, and IPS-7. Patients were excluded if they were younger than 18 years, pregnant, diagnosed at another centre and referred only for treatment without adequate baseline documentation, or had missing baseline data or insufficient follow-up to define survival outcomes. The final analytic cohort included 110 patients. Median follow-up, measured from diagnosis to last contact or death, was 39.5 months (interquartile range [IQR], 13.3–74.8 months).

### 2.2. Glasgow Prognostic Score

The GPS was assigned using established cut-offs. Patients with CRP < 1 mg/dL and albumin > 3.5 g/dL were scored as GPS 0; those with either elevated CRP (>1 mg/dL) or low albumin (<3.5 g/dL) were scored as GPS 1; and those with both abnormalities were scored as GPS 2.

### 2.3. Gustave Roussy Immune Score

The GRIm score was derived from the baseline neutrophil and lymphocyte counts, the NLR, serum albumin, and serum LDH. One point each was assigned for LDH above the upper limit of normal, albumin < 35 g/L, and NLR > 6; values within the normal range scored zero. Patients were then grouped into a low-risk category (total score 0–1) and a high-risk category (total score 2–3).

### 2.4. Endpoints

The study endpoints were treatment response, PFS, and OS. Treatment response was evaluated after first-line treatment and categorised as complete response or non-complete response; non-complete response included all categories other than complete response (partial response, inadequate/poor response, and no response). PFS was defined as the time from diagnosis to the first documented progression, relapse, or death from any cause, whichever occurred first; patients without these events were censored at last follow-up. OS was defined as the time from diagnosis to death from any cause, with surviving patients censored at last follow-up.

### 2.5. Statistical Analysis

Analyses were performed in SPSS version 25.0; Harrell’s C-index was calculated in Python 3.14.4. Python Software Foundation, Wilmington, DE, USA. Continuous variables were summarised as mean ± standard deviation or median with IQR or range, according to distribution, and categorical variables as counts and percentages. Categorical variables were compared with Pearson’s chi-square test or Fisher’s exact test, as appropriate. Treatment response was analysed as complete versus non-complete response, and binary logistic regression was used to identify factors associated with non-complete response.

Survival was estimated with the Kaplan–Meier method and compared using the log-rank test. Cox proportional hazards regression was used to estimate hazard ratios (HRs) with 95% confidence intervals (CIs) for PFS and OS. All 110 patients were included in the time-to-event analyses. Patients who were alive and event-free at last contact were treated as censored observations and contributed follow-up time up to the censoring date, rather than being removed from the models; censored cases were therefore retained in, and not excluded from, the Cox procedure. Univariable models were constructed first for GRIm, GPS, and IPS-7. To assess whether GPS and GRIm added prognostic information beyond IPS-7, additional Cox models adjusted for IPS-7 were fitted. GPS was entered as an ordinal variable, GRIm as a binary variable, and IPS-7 as a continuous score unless otherwise stated. Because the number of events was limited, particularly for OS, multivariable models were restricted to a small number of covariates to limit overfitting. Model discrimination was assessed with Harrell’s C-index for four models (IPS-7 alone; IPS-7 + GPS; IPS-7 + GRIm; and IPS-7 + GPS + GRIm), with 95% CIs estimated from 1000 bootstrap resamples. Nested Cox models were compared using likelihood-ratio (LR) tests based on changes in −2 log likelihood. A two-sided *p*-value < 0.05 was considered statistically significant.

## 3. Results

### 3.1. Patient Characteristics

The study included 110 patients with HL (median follow-up 39.5 months; IQR 13.3–74.8). Most patients were male (63.6%), and mixed cellularity was the most common subtype (53.6%). Advanced-stage disease was present in 69.1%, bulky disease in 21.8%, and B symptoms in 39.1%. Bone marrow involvement was detected by biopsy in 16.4% and by PET/CT in 39.1%. ABVD-based chemotherapy was the predominant regimen (94.5%), and complete response was achieved in 81.8%. Relapse, progression, and death occurred in 12.7%, 17.3%, and 15.5%, respectively. Baseline characteristics are summarised in Table 1.

### 3.2. Treatment Response

Overall, 90 patients (81.8%) achieved a complete response, and 20 (18.2%) had a non-complete response. Non-complete response was numerically more frequent in the GRIm high-risk group than in the low-risk group (26.3% vs. 16.5%), but the difference was not significant (*p* = 0.312). A similar non-significant gradient was seen across GPS categories (13.3%, 18.3%, and 25.0% for GPS 0, 1, and 2; *p* = 0.577). By contrast, IPS-7 was significantly associated with response: non-complete response rose from 7.8% in the low-risk group to 30.8% in the intermediate-risk group and 42.9% in the high-risk group (*p* = 0.003). The distribution of treatment response by score is shown in Table 2.

In logistic regression, IPS-7 was associated with non-complete response in univariable analysis (odds ratio [OR] = 1.85 per point, 95% CI 1.25–2.73, *p* = 0.002) and remained significant after adjustment for GPS or GRIm. Neither GPS nor GRIm was independently associated with non-complete response in adjusted models (Table 3).

### 3.3. Kaplan–Meier Survival Analyses

**Figure 1:**
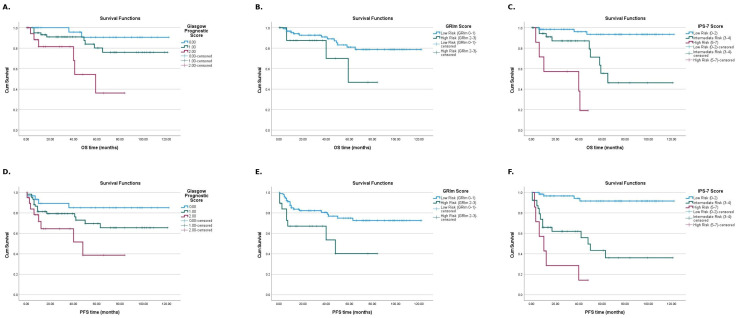
Kaplan–Meier curves for OS (**A**–**C**) and PFS (**D**–**F**) according to GPS (**A**,**D**), GRIm (**B**,**E**), and IPS-7 (**C**,**F**) categories.

During follow-up, 28 patients (25.5%) progressed or died and 17 (15.5%) died. PFS differed significantly by GRIm, GPS, and IPS-7. PFS events were more frequent in the GRIm high-risk group than the low-risk group (42.1% vs. 22.0%; log-rank *p* = 0.014) and increased across GPS categories (13.3%, 26.7%, 40.0% for GPS 0, 1, 2; *p* = 0.021). IPS-7 produced the clearest separation, with PFS event rates of 6.3%, 46.2%, and 85.7% across low-, intermediate-, and high-risk groups (*p* < 0.001). For OS, GRIm showed a numerical but non-significant difference (21.1% vs. 14.3% deaths; *p* = 0.096), whereas GPS was significantly associated with OS (6.7%, 15.0%, and 30.0% deaths for GPS 0, 1, 2; *p* = 0.003). Estimates are presented in Table 4 and survival curves in Figure 1.

### 3.4. Cox Regression Analyses

In univariable PFS models, high GRIm risk (HR = 2.683, 95% CI 1.173–6.137, *p* = 0.019), higher GPS (HR = 2.183 per point, 95% CI 1.226–3.887, *p* = 0.008), and higher IPS-7 (HR = 2.099 per point, 95% CI 1.588–2.773, *p* < 0.001) were associated with shorter PFS. After adjustment for IPS-7, neither GPS (HR = 0.665, 95% CI 0.314–1.410, *p* = 0.288) nor GRIm (HR = 1.103, 95% CI 0.457–2.661, *p* = 0.828) remained independently associated with PFS, and in the full model only IPS-7 was significant.

For OS, GPS and IPS-7 were significant in univariable analysis, whereas GRIm was not. The HR was 3.194 per point for GPS (95% CI 1.487–6.859, *p* = 0.003) and 2.671 per point for IPS-7 (95% CI 1.790–3.986, *p* < 0.001); GRIm high-risk status carried a higher but non-significant mortality hazard (HR = 2.531, 95% CI 0.812–7.891, *p* = 0.110). In IPS-7-adjusted OS models, neither GPS nor GRIm retained independent significance. All 110 patients contributed to the OS analysis; the 93 patients who were alive at last follow-up were entered as censored observations and were retained in, rather than excluded from, the Cox models. Full results are shown in Table 5.

### 3.5. Incremental Prognostic Value Beyond IPS-7

Because IPS-7 dominated the adjusted Cox models, we tested whether adding GPS or GRIm improved performance beyond IPS-7. For PFS, the IPS-7-only model had a Harrell’s C-index of 0.771 (95% CI 0.671–0.852). The C-index increased only slightly after adding GPS (0.782), GRIm (0.775), or both (0.789), and LR testing showed no significant improvement over IPS-7 alone. The same pattern held for OS: the IPS-7-only C-index of 0.805 (95% CI 0.625–0.932) rose marginally with GPS (0.819), barely changed with GRIm (0.807), and reached 0.816 for the full model, with no significant gain in model fit. These results indicate only a limited incremental value of GPS and GRIm beyond IPS-7 in this cohort. Results are summarised in Table 6.

## 4. Discussion

In this single-center cohort of 110 patients with HL, baseline GPS and GRIm were associated with PFS in univariable analyses, and GPS was also associated with OS. IPS-7 showed the strongest and most consistent association with both treatment response and survival outcomes. After adjustment for IPS-7, however, neither GPS nor GRIm retained independent prognostic significance for PFS or OS. In addition, adding GPS, GRIm, or both scores to IPS-7 resulted in only small numerical changes in Harrell’s C-index and did not significantly improve model fit on likelihood-ratio testing. These findings suggest that GPS and GRIm carry prognostic signal in univariable analyses, but their incremental value beyond IPS-7 was limited in this cohort.

The observed associations are biologically plausible. HL is characterised by a prominent inflammatory microenvironment, and systemic inflammatory activity may be reflected in routinely measured laboratory parameters. GPS is based on CRP and albumin, which represent systemic inflammation and nutritional reserve. GRIm incorporates albumin together with LDH and the neutrophil-to-lymphocyte ratio, thereby combining markers of nutritional status, tumour burden, and host inflammatory response. Higher scores may therefore identify patients with a less favourable baseline inflammatory and nutritional profile at diagnosis.

The loss of significance after adjustment for IPS-7 is also clinically understandable. Several components of IPS-7 overlap conceptually or biologically with GPS and GRIm. Albumin is directly included in IPS-7, while stage, haemoglobin level, leukocyte count, and lymphocyte count capture dimensions of tumour burden, systemic illness, and immune status. Therefore, GPS and GRIm may partly reflect adverse biology already represented within IPS-7 rather than providing a clearly independent prognostic axis. This interpretation is supported by the model performance analyses, in which the addition of GPS and/or GRIm to IPS-7 produced only marginal, non-significant improvements in discrimination.

GPS appeared more consistent than GRIm in the present cohort. GPS was associated with both PFS and OS in univariable analyses, whereas GRIm was associated with PFS but not OS. One possible explanation is that GPS is built on CRP and albumin, two direct and clinically interpretable markers of systemic inflammation and nutritional status. In contrast, GRIm uses a fixed NLR threshold, which may reduce sensitivity in HL, where lymphocyte-related variables already have established prognostic relevance and are also represented in IPS-7. The limited number of deaths in this cohort may also have reduced statistical power for detecting an association between GRIm and OS. The GPS 2 subgroup, defined by concurrent elevated CRP and hypoalbuminemia, appeared to represent the most adverse inflammatory–nutritional phenotype and may be the clinically most informative GPS category. The treatment response analysis added an important clarification. Although the non-complete response was numerically more common in patients with higher GPS or high GRIm risk, neither GPS nor GRIm was significantly associated with non-complete response. IPS-7, however, was consistently associated with treatment response in univariable and adjusted logistic regression models. Thus, the prognostic signal of GPS and GRIm in this cohort appears more evident for time-to-event outcomes than for initial treatment response.

Our findings are generally consistent with previous evidence supporting the prognostic relevance of inflammation- and nutrition-based indices in HL and other haematological malignancies. Witte et al. reported that GPS at diagnosis independently predicted survival in advanced-stage classical HL [10]. Other routine blood-based indices, including the HALP score and post-treatment neutrophil and lymphocyte counts, have also been associated with relapse and survival outcomes in HL [16,17]. In addition, composite inflammatory indices have been linked to primary refractory disease in newly diagnosed classical HL [18]. These findings support the concept that systemic inflammation and nutritional reserve are relevant to HL outcomes.

Evidence for GRIm in HL remains limited, and most available data come from solid tumour cohorts. High GRIm scores have been associated with poorer treatment response or survival in several malignancies, including breast, gastric, renal, and colorectal cancers [13,14,15,19,20]. Modified GRIm-based models have also been evaluated in advanced gastric cancer, biliary tract carcinoma, and resectable proximal gastric cancer, with consistent associations between higher inflammatory risk and worse clinical outcomes [21,22,23]. GPS has likewise shown prognostic value in diffuse large B-cell lymphoma and multiple myeloma [24,25,26]. These studies support the broader relevance of inflammatory and nutritional markers across cancer types. However, the present data indicate that prognostic signals observed in other tumours, or in univariable HL analyses, should not be assumed to provide independent value once a disease-specific model such as IPS-7 is considered.

From a practical perspective, GPS and GRIm have clear advantages. They are inexpensive, routinely available, and can be calculated from baseline laboratory tests without additional procedures or specialised imaging. Their most appropriate use may be as descriptive markers of baseline inflammatory and nutritional risk, particularly in patients with pronounced abnormalities such as GPS 2. These scores may help identify patients who require closer clinical monitoring, nutritional assessment, or careful evaluation of performance status. They may also be useful in settings where some IPS-7 components or PET-derived quantitative metrics are unavailable. However, based on the present findings, GPS and GRIm should not be considered alternatives to IPS-7, nor should they be used alone to guide treatment escalation or de-escalation. In keeping with this, GPS and GRIm are best viewed as supportive markers that require external validation before they could be considered for any role in guiding treatment decisions, rather than as independent decision-making tools.

This study has several limitations. Its retrospective, single-center design introduces potential selection bias and limits generalisability. The cohort size was modest, and the number of outcome events was limited, particularly for OS, with 28 PFS events and 17 deaths. This limited statistical power and required restriction of multivariable models to reduce the risk of overfitting. GPS and GRIm were calculated only at diagnosis, and dynamic changes during treatment could not be evaluated. CRP may also be influenced by intercurrent infection or other inflammatory conditions. Baseline PET-derived quantitative parameters, such as metabolic tumour volume, were not uniformly available and could not be incorporated into the prognostic models. In addition, the proportional hazards assumption underlying the Cox models was not formally tested with a dedicated statistical procedure; the reported hazard ratios should therefore be interpreted as average effects over the follow-up period, and this should be regarded as a further methodological limitation. Finally, although the cohort largely consisted of classical HL subtypes, the inclusion of a small number of nodular lymphocyte-predominant HL cases may limit direct comparability with studies restricted exclusively to classical HL. Larger prospective multicenter studies are needed to determine whether inflammatory and nutritional indices add clinically meaningful information when evaluated together with IPS-7 and PET-based parameters.

## 5. Conclusions

In this single-center retrospective cohort of patients with HL, baseline GPS and GRIm were associated with survival outcomes in univariable analyses, particularly for PFS, and GPS was also associated with OS. However, after adjustment for IPS-7, neither score retained independent prognostic significance, and neither meaningfully improved model discrimination or fit beyond IPS-7. GPS and GRIm are inexpensive and routinely available markers that may help characterise baseline inflammatory and nutritional risk, but they should not be interpreted as substitutes for established HL prognostic models. Their role in HL risk stratification should be clarified in larger prospective multicenter cohorts. Overall, GPS and GRIm should be regarded as supportive markers of baseline inflammatory and nutritional status that require external validation, rather than as tools that can independently guide treatment decisions.

## Figures and Tables

**Table 1:** General characteristics of the study cohort (*n* = 110).

Variable	Category	*n*	%
Sex	Male	70	63.6
	Female	40	36.4
Histopathological subtype	Nodular sclerosis	40	36.4
	Mixed cellularity	59	53.6
	Lymphocyte-rich	6	5.5
	Lymphocyte-depleted	3	2.7
	Nodular lymphocyte-predominant	2	1.8
Disease stage	Early stage (I-II)	34	30.9
	Advanced stage (III-IV)	76	69.1
Ann Arbor stage	IA	6	5.5
	IB	1	0.9
	IIA	21	19.1
	IIB	6	5.5
	IIIA	18	16.4
	IIIB	17	15.5
	IVA	22	20.0
	IVB	19	17.3
Bulky disease	Present	24	21.8
	Absent	86	78.2
B symptoms	Present	43	39.1
	Absent	67	60.9
Bone marrow involvement by biopsy	Present	18	16.4
	Absent	92	83.6
Bone marrow involvement by PET/CT	Present	43	39.1
	Absent	67	60.9
Treatment regimen	ABVD	104	94.5
	MOPP	1	0.9
	R-CHOP	2	1.8
	COPP	3	2.7
Treatment response	Complete response	90	81.8
	Partial response	7	6.4
	Stable/poor response	5	4.5
	No response	8	7.3
Relapse	Present	14	12.7
	Absent	94	85.5
	Unknown/not reported	2	1.8
Progression	Present	19	17.3
	Absent	91	82.7
Survival status	Deceased	17	15.5
	Alive	93	84.5
Comorbidities	Diabetes	11	10.0
	Hypertension	8	7.3
	Cardiac disease	4	3.6
	Thyroid disease	1	0.9
	Malignancy	2	1.8
	Other	16	14.5
	None	68	61.8

Note: PET/CT, positron emission tomography/computed tomography; ABVD, doxorubicin, bleomycin, vinblastine, dacarbazine; MOPP, mechlorethamine, vincristine, procarbazine, prednisone; R-CHOP, rituximab, cyclophosphamide, doxorubicin, vincristine, prednisone; COPP, cyclophosphamide, vincristine, procarbazine, prednisone.

**Table 2:** Association of GRIm, GPS, and IPS-7 with treatment response.

Score	Category	Complete Response, *n*/N (%)	Non-Complete Response, *n*/N (%)	*p*-Value
GRIm	Low risk	76/91 (83.5)	15/91 (16.5)	0.312
	High risk	14/19 (73.7)	5/19 (26.3)	
GPS	0	26/30 (86.7)	4/30 (13.3)	0.577
	1	49/60 (81.7)	11/60 (18.3)	
	2	15/20 (75.0)	5/20 (25.0)	
IPS-7	Low risk	59/64 (92.2)	5/64 (7.8)	0.003
	Intermediate risk	27/39 (69.2)	12/39 (30.8)	
	High risk	4/7 (57.1)	3/7 (42.9)	

Note: Non-complete response included all response categories other than complete response. *p*-values were calculated using Pearson’s chi-square test; because some expected cell counts were below 5, results should be interpreted cautiously. GRIm, Gustave Roussy Immune Score; GPS, Glasgow Prognostic Score; IPS-7, International Prognostic Score.

**Table 3:** Logistic regression analyses for non-complete response.

Model	Variable	OR	95% CI	*p*-Value
Univariable	GRIm high risk	1.810	0.566–5.782	0.317
Univariable	GPS, per 1-point increase	1.472	0.709–3.058	0.300
Univariable	IPS-7, per 1-point increase	1.845	1.249–2.726	0.002
IPS-7 + GPS	IPS-7	2.233	1.318–3.784	0.003
	GPS	0.566	0.216–1.481	0.246
IPS-7 + GRIm	IPS-7	1.916	1.245–2.948	0.003
	GRIm	0.753	0.200–2.841	0.675
IPS-7 + GPS + GRIm	IPS-7	2.245	1.312–3.840	0.003
	GPS	0.573	0.211–1.555	0.275
	GRIm	0.931	0.230–3.769	0.920

Note: The outcome variable was non-complete response. GRIm was entered as a binary variable, GPS as an ordinal variable, and IPS-7 as a continuous score. OR, odds ratio; CI, confidence interval.

**Table 4:** Kaplan–Meier survival analyses according to GRIm, GPS, and IPS-7.

Endpoint	Score	Category	Events/Total (%)	Mean Survival, Months (95% CI)	*p*-Value
PFS	GRIm	Low risk	20/91 (22.0)	94.5 (84.0–105.0)	0.014
		High risk	8/19 (42.1)	46.8 (28.7–65.0)	
PFS	GPS	0	4/30 (13.3)	106.0 (91.4–120.6)	0.021
		1	16/60 (26.7)	87.7 (73.9–101.4)	
		2	8/20 (40.0)	46.0 (27.8–64.2)	
PFS	IPS-7	Low risk	4/64 (6.3)	114.0 (106.4–121.5)	<0.001
		Intermediate	18/39 (46.2)	59.5 (40.2–78.7)	
		High risk	6/7 (85.7)	17.3 (4.4–30.1)	
OS	GRIm	Low risk	13/91 (14.3)	103.7 (94.6–112.7)	0.096
		High risk	4/19 (21.1)	60.7 (43.1–78.3)	
OS	GPS	0	2/30 (6.7)	114.5 (104.6–124.4)	0.003
		1	9/60 (15.0)	101.2 (89.5–112.8)	
		2	6/20 (30.0)	53.3 (36.3–70.3)	

Note: Survival curves were compared using the log-rank test. Mean survival is reported because median survival was not reached in several strata. PFS, progression-free survival; OS, overall survival; CI, confidence interval.

**Table 5:** Cox regression analyses for PFS and OS.

Endpoint	Model	Variable	HR	95% CI	*p*-Value
PFS	Univariable	GRIm high risk	2.683	1.173–6.137	0.019
PFS	Univariable	GPS, per 1-point	2.183	1.226–3.887	0.008
PFS	Univariable	IPS-7, per 1-point	2.099	1.588–2.773	<0.001
PFS	IPS-7 + GPS	IPS-7	2.442	1.627–3.666	<0.001
PFS	IPS-7 + GPS	GPS	0.665	0.314–1.410	0.288
PFS	IPS-7 + GRIm	IPS-7	2.076	1.544–2.791	<0.001
PFS	IPS-7 + GRIm	GRIm	1.103	0.457–2.661	0.828
PFS	IPS-7 + GPS + GRIm	IPS-7	2.445	1.623–3.683	<0.001
PFS	IPS-7 + GPS + GRIm	GPS	0.601	0.270–1.338	0.213
PFS	IPS-7 + GPS + GRIm	GRIm	1.397	0.532–3.665	0.498
OS	Univariable	GRIm high risk	2.531	0.812–7.891	0.110
OS	Univariable	GPS, per 1-point	3.194	1.487–6.859	0.003
OS	Univariable	IPS-7, per 1-point	2.671	1.790–3.986	<0.001
OS	IPS-7 + GPS	IPS-7	2.966	1.677–5.246	<0.001
OS	IPS-7 + GPS	GPS	0.772	0.294–2.027	0.599
OS	IPS-7 + GRIm	IPS-7	2.763	1.818–4.198	<0.001
OS	IPS-7 + GRIm	GRIm	0.743	0.224–2.463	0.627
OS	IPS-7 + GPS + GRIm	IPS-7	2.956	1.677–5.209	<0.001
OS	IPS-7 + GPS + GRIm	GPS	0.825	0.288–2.364	0.720
OS	IPS-7 + GPS + GRIm	GRIm	0.813	0.220–3.004	0.756

Note: Models were kept restricted because of the limited number of events, particularly for OS. GPS was entered as an ordinal variable, GRIm as a binary variable, and IPS-7 as a continuous score unless otherwise specified. HR, hazard ratio; CI, confidence interval; PFS, progression-free survival; OS, overall survival.

**Table 6:** Incremental prognostic value of GPS and GRIm beyond IPS-7.

Endpoint	Model	−2LL	LR χ^2^ vs. IPS-7	df	*p*-Value	C-Index (95% CI)
PFS	IPS-7 alone	216.045	Reference	–	–	0.771 (0.671–0.852)
PFS	IPS-7 + GPS	214.910	1.135	1	0.287	0.782 (0.681–0.862)
PFS	IPS-7 + GRIm	215.998	0.047	1	0.828	0.775 (0.672–0.857)
PFS	IPS-7 + GPS + GRIm	214.459	1.586	2	0.452	0.789 (0.684–0.874)
OS	IPS-7 alone	115.773	Reference	–	–	0.805 (0.625–0.932)
OS	IPS-7 + GPS	115.498	0.275	1	0.600	0.819 (0.637–0.940)
OS	IPS-7 + GRIm	115.528	0.245	1	0.621	0.807 (0.629–0.935)
OS	IPS-7 + GPS + GRIm	115.400	0.373	2	0.830	0.816 (0.638–0.939)

Note: LR tests compared each expanded model with the IPS-7-only model. C-index values are Harrell’s concordance index with bootstrap-derived 95% CIs. −2LL, minus two log likelihood; LR, likelihood ratio; CI, confidence interval.

## Data Availability

The data presented in this study are available on request from the corresponding author. The data are not publicly available owing to privacy and ethical restrictions.

## Abbreviations

ABVD, doxorubicin, bleomycin, vinblastine, dacarbazine; CI, confidence interval; CRP, C-reactive protein; DLBCL, diffuse large B-cell lymphoma; GPS, Glasgow Prognostic Score; GRIm, Gustave Roussy Immune Score; HALP, haemoglobin–albumin–lymphocyte–platelet; HL, Hodgkin lymphoma; HR, hazard ratio; IPS-7, seven-factor International Prognostic Score; LDH, lactate dehydrogenase; LR, likelihood ratio; NLR, neutrophil-to-lymphocyte ratio; OS, overall survival; PET/CT, positron emission tomography/computed tomography; PFS, progression-free survival.
